# Short-Term and Lifelong Exercise Training Lowers Inflammatory Mediators in Older Men

**DOI:** 10.3389/fphys.2021.702248

**Published:** 2021-08-20

**Authors:** Lawrence D. Hayes, Peter Herbert, Nicholas F. Sculthorpe, Fergal M. Grace

**Affiliations:** ^1^School of Health and Life Sciences, University of the West of Scotland, Glasgow, United Kingdom; ^2^School of Sport, Health and Outdoor Education, University of Wales Trinity Saint David, Carmarthen, United Kingdom; ^3^Faculty of Health, Federation University, Ballarat, VIC, Australia

**Keywords:** Aging, C-reactive protein, high-intensity interval training, homocysteine, interleukin-6, inflammaging, inflammation

## Abstract

Increased basal low-grade inflammation is observed with advancing age, which is augmented by physical inactivity. However, data regarding the influence of lifelong exercise training and particularly high-intensity interval training (HIIT) on inflammatory mediators in older men are scarce. Therefore, we examined effects of 6weeks of aerobic preconditioning followed by 6weeks of HIIT on inflammatory mediators [interleukin (IL)-6, homocysteine, and high-sensitivity C-reactive protein (hsCRP)] in previously sedentary older men (SED) and masters athletes (LEX). Further, we investigated whether SED exhibited greater basal inflammatory biomarkers compared to LEX. Twenty-two men (aged 62±2years) participated in the SED group, while 17 age-matched LEX men (aged 60±5years) also participated as a positive comparison group. In SED, preconditioning (*P*=0.030, *d*=0.34) and HIIT (*P*=0.030, *d*=0.48) caused a reduction in IL-6 compared to enrollment. SED homocysteine did not change throughout (*P*>0.57; *d*<0.26), while the decrease in hsCRP after preconditioning (*P*=0.486, *d*=0.25) and after HIIT (*P*=0.781, *d*=0.23) compared to enrollment was small. HIIT did not influence IL-6 or hsCRP in LEX (all *P*>0.42; *d*<0.3). Homocysteine increased from enrollment to post-HIIT in LEX (*P*=0.144, *d*=0.83), but all other perturbations were trivial. IL-6 and hsCRP were greater in SED than LEX throughout the investigation (all *P*<0.029; *d>*0.72), but homocysteine was not different (all *P* >0.131; *d*<0.41). Results of this study suggest moderate-intensity aerobic exercise and HIIT lowers IL-6 (and possible hsCRP) in previously sedentary older men. Moreover, lifelong exercise is associated with reduced concentrations of some inflammatory biomarkers in older males, and therefore, physical activity, rather than age *per se*, is implicated in chronic low-grade inflammation. Moreover, physical inactivity-induced inflammation may be partly salvaged by short-term exercise training.

## Introduction

Increased basal inflammation is generally considered a side effect of poor health and an unhealthy lifestyle (Beavers et al., 2010; Nimmo et al., 2013; Papadopoulos and Santa Mina, 2018). Moreover, the increased concentrations of pro-inflammatory cytokines are associated with several noncommunicable diseases, such as cardiovascular disease (Ramage and Guy, 2004), diabetes (Qu et al., 2014), cancer (Papadopoulos and Santa Mina, 2018), and obesity (Monzillo et al., 2003; Accattato et al., 2017). The significance of chronic low-grade inflammation in disease risk is compelling. For example, there is evidence for high-sensitivity C-reactive protein (hsCRP; whose level rises in response to inflammation) as a strong independent predictor of cardiovascular disease (Ridker et al., 1997, 1998; Pai et al., 2004; Ridker, 2004). Likewise, the Rancho Bernardo Study of Healthy Aging demonstrated that for each standard deviation (SD) increase in interleukin (IL)-6, lifespan was 1year less, underlining the importance of inflammatory cytokines in longevity (Wassel et al., 2010; Lee et al., 2012).

Systemic inflammation typically increases with age (termed *inflammaging*; Franceschi et al., 2000), and circulating cytokine dysregulation is a well-recognized result of biological aging (Álvarez-Rodríguez et al., 2012). The inflammaging hypothesis proposes aging increases reactive oxygen species and leads to a more pro-inflammatory basal state (Franceschi et al., 2007). For example, tumor necrosis factor alpha (TNF-α) has been reported to be increased in a stepwise manner, with centenarians displaying greater concentrations than 80-year-olds, who in turn display greater concentrations than younger individuals (Franceschi et al., 2007). Similarly, homocysteine is greater in those aged over 50years of age compared to younger individuals and is associated with unhealthy lifestyle habits, such as smoking (Xu et al., 2020). Moreover, IL-6 is greater in advanced age (Bruunsgaard et al., 1999; Baylis et al., 2013), while intracellular pro-inflammatory cytokines, such as interferon gamma (IFN-γ) and TNF-α, are greater in older participants’ T cells than younger counterparts’ T cells (Zanni et al., 2003).

This increased pro-inflammatory state is ameliorated by physical activity (Nimmo et al., 2013; Hennigar et al., 2017; Cabanas-Sánchez et al., 2018). It is thought that exercise exerts a direct anti-inflammatory effect, but also alleviation of adipose-induced inflammation (Beavers et al., 2010). In support of this, Taaffe et al. (2000) reported that IL-6 and CRP were inversely related with hours per year of moderate and strenuous physical activity. Moreover, physical function measured by walking speed and grip strength was inversely correlated with IL-6 and CRP in septuagenarians (Taaffe et al., 2000). Based on the available literature, aerobic exercise training appears more likely to reduce inflammation than resistance training (Nicklas et al., 2008; Beavers et al., 2010). Moreover, both greater frequency and greater intensity of aerobic training enhance anti-inflammatory effects (Hsu et al., 2009; Hawkins et al., 2012). In a recent systematic review covering 2016–2020, consistent anti-inflammatory effects of exercise included lowering of CRP, IL-6, and TNF-α (Bautmans et al., 2021), mirroring the findings of an earlier review by the same authors (Liberman et al., 2017). One striking outcome of these systematic reviews, however, was that resistance exercise was the most examined exercise type, followed by aerobic conditioning, then a combination of the two, and finally “other” which consisted of tai chi, Pilates, and balance training. The reason this observation is of note is that despite interventions incorporating resistance training being most prevalent, this is not reflected in the number of people adhering to the public health guidelines regarding muscle strengthening exercise (Strain et al., 2016). In fact, in those >70years of age, fewer than 98% of people complete twice weekly muscle strengthening exercises (Strain et al., 2016). In similar fashion, none of the studies included within these meta-analyses utilized a high-intensity interval training (HIIT) intervention.

Given that higher exercise intensity is reportedly more efficacious in reducing inflammatory mediators, it would be prudent to consider HIIT as a viable alternative to moderate-intensity continuous exercise and/or resistance exercise to reduce chronic low-grade inflammation. HIIT is considered healthogenic across several physiological systems (Gillen and Gibala, 2014; Weston et al., 2014; Grace et al., 2015; Knowles et al., 2015; Ramos et al., 2015), yet there are scarce data concerning HIIT-induced alterations to inflammatory cytokines and mediators in older adults. Taken together, the aims of this study were: (a) to examine effects of HIIT preceded by preconditioning on IL-6, homocysteine, and hsCRP in previously sedentary older men (SED); (b) to examine the impact of HIIT (and simultaneous reduction of other habitual training) on IL-6, homocysteine, and hsCRP in lifelong exercising masters athletes (LEX); and (c) to test whether LEX exhibited lower concentrations of inflammatory mediators than age-matched SED men. Our *a priori* hypotheses were as follows: (a) Six weeks of HIIT following preconditioning exercise would reduce IL-6, homocysteine, and hsCRP in SED men; (b) LEX would exhibit no alteration to these inflammatory mediators following HIIT; and (c) IL-6, homocysteine, and hsCRP would be greater in SED than LEX.

## Materials and Methods

### Participants

**Figure 1:**
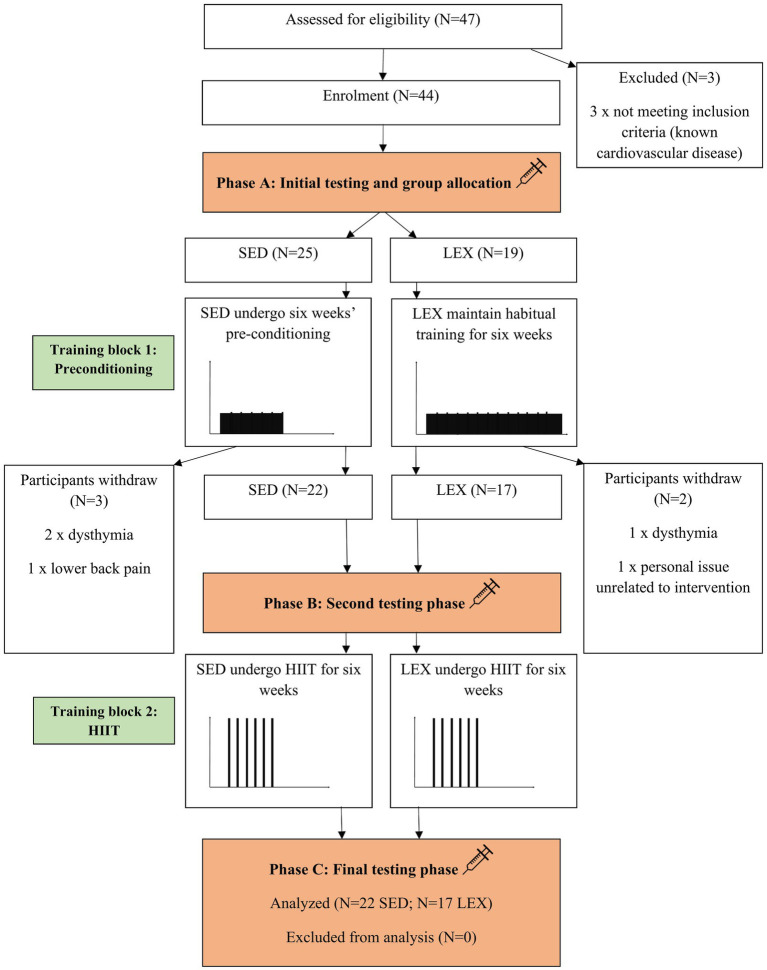
The Consolidated Standards of Reporting Trials (CONSORT) flow diagram detailing the experimental process in which lifelong exercising (LEX) and lifelong sedentary (SED) men completed the intervention. HIIT=high-intensity interval training.

Following ethical approval by the Institutional Ethics Committee and written informed consent, 22 volunteered and were included in the SED group [62±2years of age, with a mass of 91±16kg, stature of 175±6cm, and peak oxygen uptake (VO_2peak_) of 28±6mlkgmin^−1^] and 17 men participated as LEX (60±5years of age, with a mass of 78±12kg, stature of 173±6cm, and VO_2peak_ of 39±6mlkgmin^−1^). The SED group had not participated in exercise programs for over 30years. Conversely, LEX participants had all been training for >30years and competed in masters events, such as triathlon, water-polo, road cycling, track cycling, and distance running. One of the authors (PH) questioned participants in advance of the study, and the majority of LEX included some infrequent, generally isolated high-intensity efforts which were not in an organized manner (i.e., cannot constitute interval training *per se*). Therefore, despite being well trained, LEX participants were considered HIIT-naïve at enrollment. On testing days, participants reported to the laboratory between 07:00 and 09:00h after an overnight fast and had not exercised for >48h. Similarly, participants had not drunk alcohol or caffeine for a minimum of 36h. As a condition to study enrollment, general medical practitioners (GPs) for each potential participant were contacted and provided with a copy of the study design, protocols, and intended exercise programs. GPs were invited to contact the investigators with any query relating to the study and were further required to provide a written letter of approval for their patient to enroll to the study. Participants were withdrawn if, in the opinion of their GP, risks to their health were present. This could include a history of myocardial infarction, angina, stroke, and chronic pulmonary disease. Consequently, three of the original 47 participants withdrew under GP advice (Figure 1). Four of the SED group were on antihypertensive medication according to their physical activity readiness questionnaire, and all remaining participants indicated “nil” to the question concerning medication.

### Blood Collection and Analysis

Venous blood samples were collected from an antecubital vein at each phase between 07:00 and 09:00h, 48–72h following the last exercise session, by the same phlebotomist, as previously described (Hayes et al., 2017, 2020; Herbert et al., 2017a). Serum IL-6, homocysteine, and hsCRP were determined by electrochemiluminescent immunoassay (E601 module of the Roche Cobas 6000, Burgess Hill, West Sussex, United Kingdom) in the Clinical Biochemistry Laboratory at Royal Glamorgan Hospital (Wales, United Kingdom). Coefficients of variations (CV) over 6months were all <5%.

### Body Composition and Physical Fitness

Bioelectrical impedance analysis (Tanita MC-180MA Body Composition Analyzer, Tanita UK Ltd.) as used to determine body composition (i.e., lean body mass, fat mass, and body fat percentage). VO_2peak_ was measured using breath-by-breath gas analysis (Cortex II Metalyser 3B-R2, Cortex, Biophysik, Leipzig, Germany) using a modified Storer Test (Storer et al., 1990), as reported previously (Knowles et al., 2015). The 6s Herbert test (Herbert et al., 2015) was used to determine peak power output (PPO) on a cycle ergometer (Wattbike Pro, Wattbike Ltd., Nottingham, United Kingdom). Order of measurement was blood draws, body composition, PPO, and VO_2peak_. All details have been previously described (Grace et al., 2015).

### Exercise Training

Two six-week training blocks separated the three testing phases (phase A, B, and C). The full protocol has been previously reported by Grace et al. (2015), so training is detailed here briefly to avoid replication. During training block 1 (between testing phases A and B), SED underwent the physical activity guidelines of moderate to vigorous aerobic exercise for 150min wk^−1^ (Riebe et al., 2015) of which was recorded through heart rate telemetry (Polar FT1, Polar, Kempele, Finland). During this time, LEX continued their habitual training which we monitored by heart rate telemetry. During training block 2 (between testing phases B and C), both groups underwent a HIIT program. Sessions consisted of efforts at 40% PPO for 30s with 3min recovery between each interval. Frequency of training was once every five days (i.e., nine HIIT sessions in total).

### Statistical Analysis

Data analysis was completed with Jamovi version 1.6.23.0. Following confirmation of normal distribution (Shapiro-Wilk test) and homogeneity of variance (Levene’s test), 2×3 [group (SED, LEX)×time (phase A, B, C)] mixed factorial ANOVAs were conducted to test for differences in concentrations of IL-6, homocysteine, and hsCRP between groups and time points. Subsequently, one-way ANOVA with *post-hoc* paired *T*-tests with Bonferroni corrections were performed to locate differences between phases A, B, and C in each group. As the Bonferroni correction can be simplified to $\frac{\alpha }{n}$; where α is the set probability threshold and *n* is the number of comparisons, we report P values from each *T*-test multiplied by the number of comparisons [three for within-group between-phase comparisons (i.e., SED A vs. SED B vs. SED C)]. Moreover, *posteriori* independent samples *T*-tests were performed to locate differences between LEX and SED at a particular test phase, without correcting for the number of comparisons [as there was only one (i.e., SED A vs. LEX A)]. As adiposity is known to influence inflammatory biomarkers, analyses of covariance (ANCOVAs) were performed on inflammatory biomarkers at each phase to locate differences between SED and LEX with body fat percentage as the covariate. Correlations between variables were examined with Pearson’s correlation coefficient. Alpha levels are reported as exact P values, and we do not defined P values as “significant” or “non-significant” as advised by the American Statistical Association (Hurlbert et al., 2019). Effect sizes are reported using Cohen’s *d* and classified using thresholds specific to gerontology (Brydges, 2019) which are 0.15≥small, 0.40≥moderate, and 0.75≥large. Figures are shown as grouped dot plots with mean and SD as recommended by Drummond and Vowler (2012), but also dot plots with connected lines to show individual responses to the intervention. Data are presented as mean±SD [95% confidence intervals (CI)].

## Results

Adherence to the intervention was 100%.

### Interleukin-6

**Figure 2:**
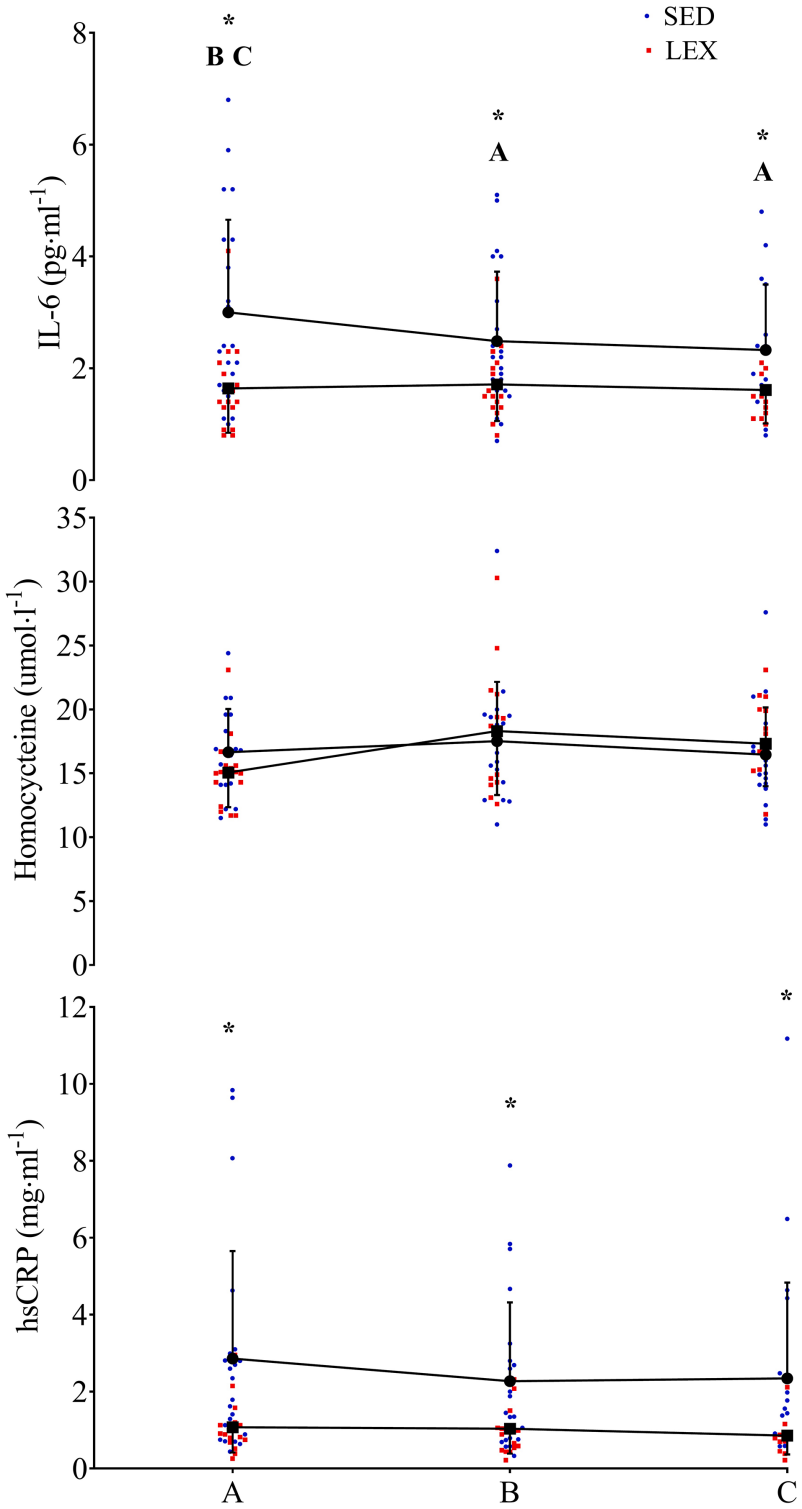
IL-6 (top), homocysteine (middle), and high-sensitivity C-reactive protein (hsCRP; bottom) in a group of previously sedentary (SED) older men and LEX older men. Data are presented as mean±SD, plus individual data points. ^*^Group differences at this experimental phase at the *P*<0.05 level. *A*=SED group difference from phase A at the *P*<0.05 level. *B*=SED group difference from phase B at the *P*<0.05 level. *C*=SED group difference from phase C at the *P*<0.05 level.

**Figure 3:**
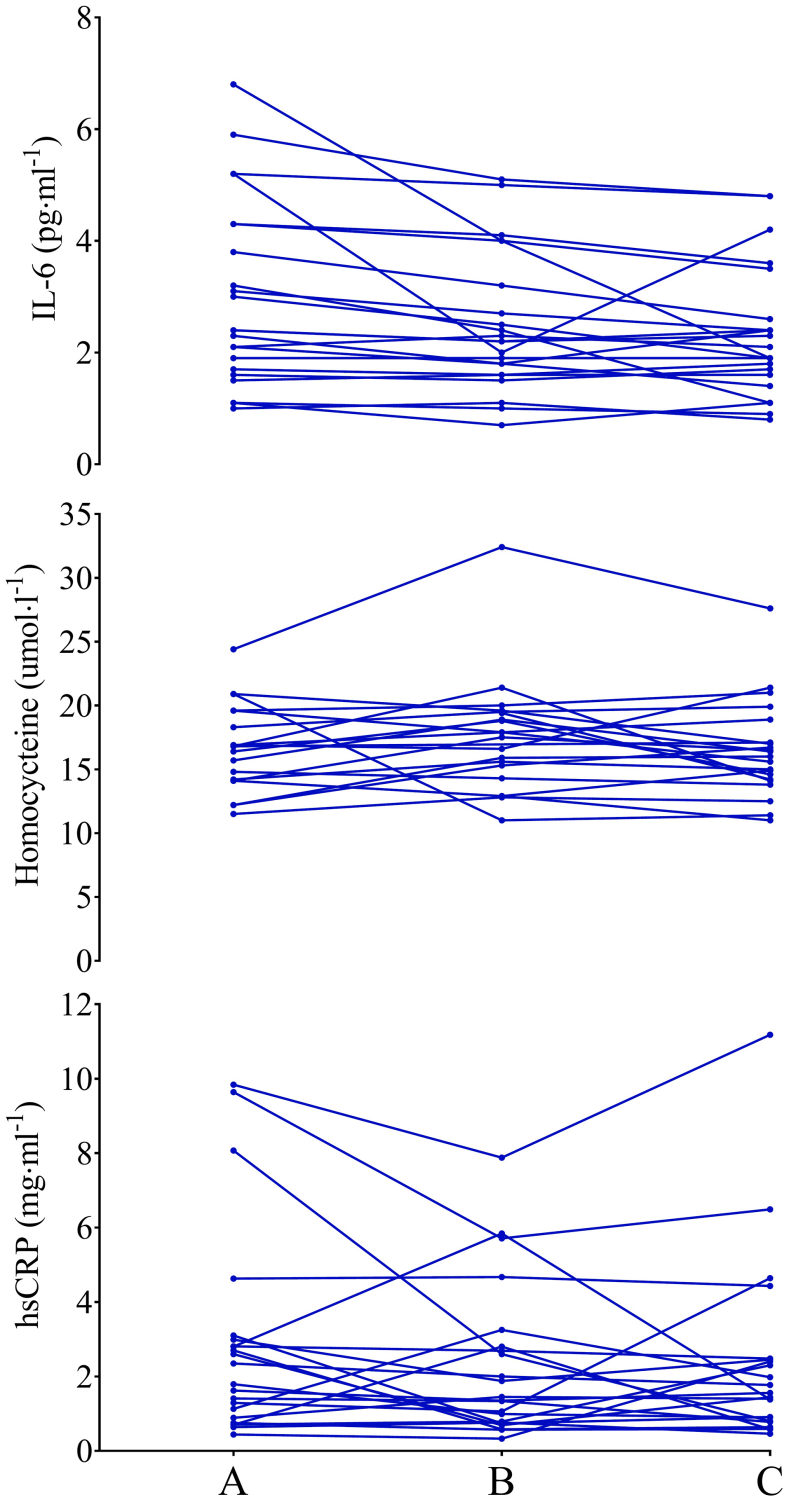
IL-6 (top), homocysteine (middle), and hsCRP (bottom) in previously sedentary older men. Data are presented as individual data points to visualize individual responses.

**Figure 4:**
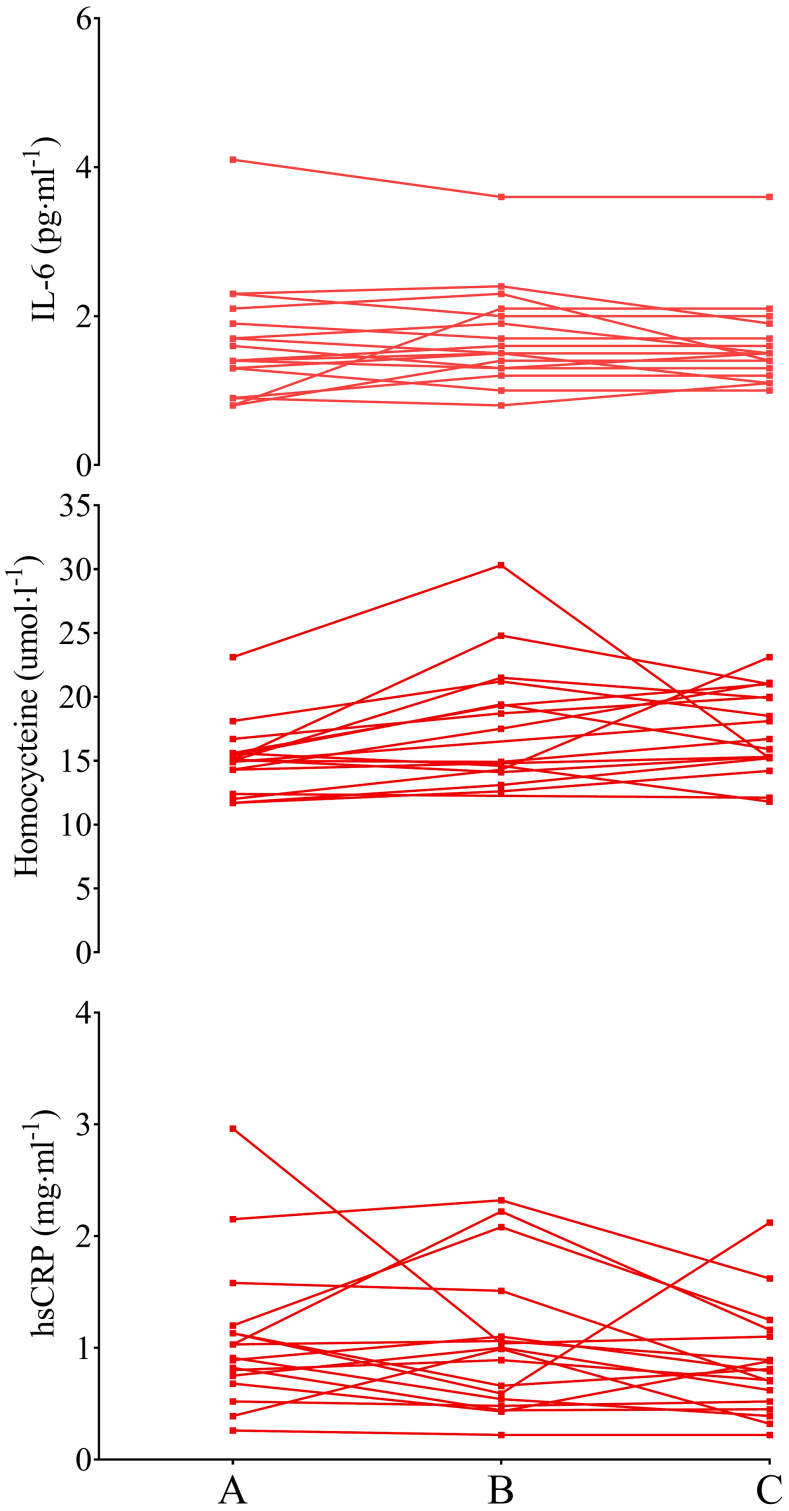
IL-6 (top), homocysteine (middle), and hsCRP (bottom) in LEX older men. Data are presented as individual data points to visualize individual responses. Note, the y-axis for IL-6 and hsCRP has been compressed compared to Figures 2, 3.

The mixed factorial ANOVA concerning IL-6 resulted in a main effect of time (*P*=0.017), group (*P*=0.008), and interaction between time and group (*P*=0.017). SED IL-6 was 3.0±1.7 (95% CI 2.4–3.6), 2.5±1.2 (95% CI 2.0–2.9), and 2.3±1.2 (95% CI 1.9–2.8) pgml^−1^ at phase A, B, and C, respectively (A vs. B: *P*=0.030, *d*=0.34, B vs. C: *P*=1.000, *d*=0.17, A vs. C: *P*=0.030, *d*=0.48). LEX IL-6 was 1.6±0.8 (95% CI 1.0–2.3), 1.7±0.7 (95% CI 1.2–2.2), and 1.6±0.6 (95% CI 1.1–2.1) pgml^−1^ at phases A, B, and C, respectively (A vs. B: *P*=1.000, *d*=0.13, B vs. C: *P*=0.520, *d*=0.15, A vs. C: *P*=1.000, *d*=0.02). Thus, IL-6 concentrations were greater in SED than LEX at phase A (*P*=0.004, *d*=1.05), B (*P*=0.026, *d*=0.81), and C (*P*=0.028, *d*=0.74; Figures 2–4). Controlling for body fat percentage with ANCOVA did not have a large effect on between group P or *d* values (phase A: *P*=0.039, *d*=0.75, phase B: *P*=0.041, *d*=0.73, Phase C: *P*=0.038, *d*=0.75).

### Homocysteine

The mixed factorial ANOVA concerning homocysteine resulted in time effect of *P*=0.171, group effect of *P*=0.816, and interaction effect of *P*=0.339. SED homocysteine was 16.6±3.6 (95% CI 15.0–18.2), 17.7±4.8 (95% CI 14.8–20.5), and 16.7±3.9 (95% CI 15.0–18.5) μmolL^−1^ at phases A, B, and C, respectively (A vs. B: *P*=0.806, *d*=0.26, B vs. C: *P*=0.573, *d*=0.23, A vs. C: *P*=1.000, *d*=0.26). LEX homocysteine was 15.3±2.8 (95% CI 13.6–17.0), 17.1±6.8 (95% CI 14.0–20.1), and 17.8±3.2 (95% CI 15.9–19.7) μmolL^−1^ at phases A, B, and C, respectively (A vs. B: *P*=0.680, *d*=0.35, B vs. C: *P*=1.000, *d*=0.13, A vs. C: *P*=0.144, *d*=0.83). Homocysteine between LEX and SED was similar throughout the study (phase A: *P*=0.132, *d*=0.40, phase B: *P*=0.830, *d*=0.10, phase C: *P*=0.457, *d*=0.31). Applying ANCOVA with body fat percentage as the covariate did not have a large effect on between group P or *d* values (phase A: *P*=0.243, *d*=0.44, phase B: *P*=0.960, *d=*0.02, phase C: *P*=0.181, *d=*0.47).

### High-Sensitivity C-reactive Protein

The mixed factorial ANOVA resulted in a time effect of *P*=0.267, a group effect of *P*=0.009, and an interaction effect of *P*=0.526 for hsCRP. In SED, hsCRP was 2.9±2.8 (95% CI 1.9–3.8), 2.3±2.0 (95% CI 1.6–3.0), and 2.3±2.5 (95% CI 1.5–3.2) mg·ml^−1^ at phases A, B, and C, respectively (A vs. B: *P*=0.486, *d*=0.25, B vs. C: *P*=1.000, *d*=0.01, A vs. C: *P*=0.781, *d*=0.23). LEX hsCRP was 1.1±0.7 (95% CI 0.02–2.1), 1.0±0.6 (95% CI 0.2–1.8), and 0.9±0.5 (95% CI 0.0–1.8) mg ml^−1^ at phases A, B, and C, respectively (A vs. B: *P*=1.000, *d=*0.15, B vs. C: *P*=0.720, *d=*0.18, A vs. C: *P*=0.425, *d*=0.33). Thus, hsCRP was lower in LEX than SED at phases A (*P*=0.014, *d=*0.88), B (*P*=0.022, *d=*0.88), and C (*P*=0.021, *d=*0.79). Controlling for body fat percentage using ANCOVA increased the P value and reduced effect size for differences between LEX and SED at phases A (*P*=0.268, *d=*0.40), B (*P*=0.205, *d=*0.44), and C (*P*=0.303, *d=*0.36).

### Correlative Analysis

IL-6 was correlated with hsCRP at phases A, B, and C (all *P*<0.009; *r*=0.413–0.835). Moreover, IL-6 concentrations were correlated with fat mass and body fat percentage at phase A (*P*<0.001; *r*=0.531 and *P*<0.001; *r*=0.456, respectively). However, this relationship did not exist at phase B or C (*P*>0.224; *r*<0.199). IL-6 was correlated with VO_2peak_ at phase A (*P*=0.006; *r*=−0.430), but not B (*P*=0.260; *r*=−0.185) or C (*P*=0.445; *r*=−0.126). hsCRP concentrations were associated with total fat mass and body fat percentage at phases A (*P*<0.001; *r*=0.744 and *P*<0.001; *r*=0.665, respectively), B (*P*<0.001; *r*=0.632 and *P*<0.001; *r*=0.606, respectively), and C (*P*<0.001; *r*=0.732 and *P*<0.001; *r*=0.678, respectively). hsCRP was correlated with VO_2peak_ at phase A (*P*=0.002; *r*=−0.488), phase B (*P*=0.003; *r*=−0.459), and phase C (*P*<0.001; *r*=−0.570). Homocysteine was not related to any body composition or fitness parameter at any phase.

The change in (∆) IL-6 (i.e., phase C IL-6 – phase A IL-6) was not associated with ∆hsCRP (*P*=0.074; *r*=0.289), ∆homocysteine (*P*=0.789; *r*=0.044), ∆body fat percentage (*P*=0.608; *r*=−0.085), or ∆VO_2peak_ (*P*=0.309; *r*=−0.167). ∆IL-6 was most strongly correlated with IL-6 at phase A (*P*<0.001; *r*=−0.742). ∆hsCRP was not associated with ∆homocysteine (*P*=0.725; *r*=0.061), ∆body fat percentage (*P*=0.441; *r*=−0.127), or ∆VO_2peak_ (*P*=0.412; *r*=−0.135). ∆hsCRP was most strongly correlated with hsCRP at phase A (*P*<0.001; *r*=−0.513). ∆homocysteine was not associated with ∆body fat percentage (*P*=0.133; *r*=0.255), or ∆VO_2peak_ (*P*=0.712; *r*=0.064). ∆homocysteine was strongly correlated with homocysteine at phase A (*P*<0.001; *r*=−0.533) and homocysteine at phase C (*P*<0.001; *r*=0.648).

## Discussion

The primary finding from the present investigation was that a period of preconditioning produced a moderate reduction in IL-6 and a small reduction in hsCRP, without change to homocysteine in SED. Moreover, HIIT maintained lower concentrations of IL-6 and hsCRP, despite the reduced training volume in SED. No alterations to these inflammatory mediators were seen in LEX, likely due to their low initial concentrations at baseline (the ceiling effect). Finally, LEX had lower concentrations of IL-6 and hsCRP, but not homocysteine, than SED throughout the investigation. However, when controlling for body fat percentage, the difference in hsCRP was attenuated. These data suggest lifetime exercise training habits are anti-inflammatory, in part through reduced adiposity, and a short period of HIIT after aerobic conditioning can produce moderate reductions in IL-6 and small reductions in hsCRP in previously sedentary older men. These data may also suggest homocysteine is not a viable target for exercise interventions.

Here, we report aerobic conditioning reduced IL-6 in lifelong sedentary older adults, which was maintained following the transition to HIIT despite the very low training volume. Interestingly, ∆IL-6 was not related to change in body composition, suggesting exercise exerted a direct effect on IL-6 rather than an indirect effect mediated by reduced adiposity. As ∆IL-6 was mostly strongly related to IL-6 at phase A (in both groups, and combined), this suggests individuals with the highest concentrations at enrollment experienced the greatest benefit, which could be interpreted as those who had the greatest IL-6 at enrollment had the most “potential” to improve. This corroborates several investigations which show those with the poorest values at baseline have the most “potential” for improvement across several physiological parameters (Little et al., 2011; Herbert et al., 2017b; Hayes et al., 2020). Our finding of improved IL-6 in SED post-training is in line with other intervention studies in older adults following adoption of exercise or physical activity (Kohut et al., 2006; Nicklas et al., 2008). For example, Nicklas and colleagues observed a decrease in IL-6 of 0.53±4.19pgml^−1^ in older adults following a 12-month physical activity intervention. In the present investigation, we report a larger magnitude of change following our 12-week exercise intervention [Cohen’s *d=*0.48 for phase A vs. C in the present study; Cohen’s *d=*0.15 for baseline vs. 12months in Nicklas et al. (2008)]. An elucidation of greater effect size seen in the present investigation could be the exercise was of a greater intensity than used in Nicklas et al. (2008) investigation, which focused on walking with a targeted perceived exertion of 1,116 out of 20. Conversely, HIIT by definition is high intensity and muscle glycogen is depleted in an intensity-dependent manner, and IL-6 is dependent on muscle glycogen availability (Keller et al., 2001; Steensberg et al., 2001; Chan et al., 2004).

In reporting lower concentrations of inflammatory mediators in a trained group of older adults (Cohen’s *d* >0.75; large effect for IL-6 and hsCRP), our findings support those of Aguiar et al. (2020), who reported middle-age masters athletes had lower IL-6 and TNF-α than an age-matched control group. These findings were observed in participants ~46–52year of age, and the present study extends these findings into sexagenarians (termed the “young-old”), supporting the contention that exercise habits modify inflammation, potentially through indirect adipose tissue reduction. In terms of inflammation and physical function correlates, Santos Morais Junior et al. (2020) observed IL-6 was correlated with frailty in very old adults which adds weight to the notion increased inflammatory mediators are consistently associated with frailty and mortality in the very old (Michaud et al., 2013; Chen et al., 2014). This investigation extends these findings to the young old, suggesting inflammatory mediators are associated with physical function, as evidenced by the correlation between IL-6 and VO_2peak_ at baseline, and hsCRP and VO_2peak_ throughout the investigation. The differences between SED and LEX at baseline also suggest lifelong exercise may be protective against inflammatory markers known to associate with frailty, in addition to the direct anti-frailty effect (Theou et al., 2011; Viña et al., 2016; da Silva et al., 2019; Oliveira et al., 2020).

Data in this study, primarily the relationships between IL-6 and hsCRP with fat mass and body fat percentage, suggest adiposity influenced initial concentrations of inflammatory mediators. Interestingly, the changes in fitness and fatness were not related to changes in inflammatory mediators in the present investigation. We suggest this may due to different temporal adaptations in these systems (i.e., cardiorespiratory vs. immune), and a longitudinal study may be required to confirm these relationships over a longer duration.

Data from this study are supportive of a recent article which suggested inflammation is physical inactivity driven, rather than a factor of age *per se* (Yasar et al., 2021). These authors examined multiple pro- and anti-inflammatory cytokines in aerobically trained older and younger males. Interestingly, of the 12 cytokines studied [epidermal growth factor (EGF), IL-1a, -1b, -2, -4, -6, -8, and -10, IFN-γ, monocyte chemoattractant protein-1, TNFα, and vascular endothelial growth factor], only EGF was different between the young and old cohort. With reference to the biomarkers measured in this study, Yasar et al. (2021) noted IL-6 was similar between young and old trained groups. We believe this is supportive of data from the present investigation as we observed differences in IL-6 between men of the same age, but divergent lifetime exercise habits, suggesting exercise rather than age influence IL-6. Likewise, Yasar et al. (2021) noted similar IL-6 in participants with an age difference of ~40years, but similar exercise habits. This leads us to agree with several authors’ conclusions that *inflammaging* is partly inactivity-driven, and not exclusively a consequence of chronological aging (Minuzzi et al., 2017, 2019; Yasar et al., 2021). Interestingly, however, Minuzzi et al. (2019) did not observe differences in IL-6 or TNFα between masters athletes and age-matched healthy middle-aged controls. This could suggest the physical inactivity-induced pro-inflammatory environment may only manifest later in life (i.e., in the old, rather than middle aged). This contention is supported by the same authors observing no difference between the middle-aged groups and a young cohort in basal IL-6 or TNFα. However, on further examination of the complete data set of Minuzzi et al. (2019), these authors did observe differences in IL-1ra, IL-1β, IL-4, and IL-8 between masters athletes and age-matched controls and the young group, causing these authors to suggest lifelong training improves the anti-inflammatory environment, which also supports their previous work (Minuzzi et al., 2017). Taken together, future research could consider both pro- and anti-inflammatory biomarkers to examine the effects of age and exercise on inflammation more comprehensively, although this would require significant resource commitment.

The present investigation is not without limitations, which we accept. Firstly, it is impossible to ascribe positive adaptations in SED to HIIT alone, but a combination of training block 1 (preconditioning) and training block 2 (HIIT) as a result of our study design. We made the decision to structure the exercise intervention this way considering the ACSM guidelines for exercise for older adults (Riebe et al., 2015) and considered it safety-conscious to precede HIIT with preconditioning. Secondly, it would have been interesting to include a trained and sedentary younger cohort to examine the age and training interplay more comprehensively. Moreover, a non-exercise control arm would have added clarity to this interaction. However, this would have necessitated greater resource commitments which were outside the scope of this project. Penultimately, the cross-sectional comparison of SED and LEX as baseline limits the conclusion that training habits rather than age drive inflammation. While LEX and SED exercise habits during adulthood were known, inflammatory biomarkers were only quantified at one age. Thus, we were unable to determine whether LEX inflammatory biomarkers were unchanged throughout adulthood or whether a gradual increase occurred irrespective of LEX/SED status, but LEX to a lesser extent than SED. Although the data from Yasar et al. (2021) suggested young (~28years of age) and old (~68years of age) trained individuals’ inflammatory biomarkers were similar and thus support our supposition, these data were also a comparison made at one time point. Therefore, serial sampling throughout adulthood (i.e., a longitudinal cohort study) would be required to determine whether training habits rather than age are entirely responsible for levels of inflammation. The greatest limitation however was that inflammatory mediators measured in this study were secondary outcomes of a previous study (Knowles et al., 2015), which used VO_2peak_ for sample size calculations. In this context, an *a posteriori* power analysis with SED IL-6 at phases A and C, and an alpha level of 0.05 resulted in statistical power of 0.52 for a one-sided test. With an alpha level of 0.1, proposed as a suitable compromise between risk of type I and type II error (Nio et al., 2017), observed power was 0.66. For homocysteine and hsCRP, using the highest and lowest concentrations observed (thus, generating the greatest statistical power), observed power was even lower.

Despite the limitations of this study, there are numerous strengths which we feel obliged to emphasize. Firstly, our use of ECLA, rather than cytokine array, ensured suitable sensitivity for the inflammatory markers investigated. This was crucial as LEX exhibited low concentrations of pro-inflammatory cytokines, which would not be detected by biochip assays used to detect clinically relevant concentrations of these cytokines. Secondly, incorporating a SED and a LEX group allowed us to compare effect of lifelong exercise habits vs. sedentarism (LEX vs. SED), and the effects of HIIT in sedentary and lifelong exercising (but HIIT naïve) participants. In this context, results presented here are encouraging, as they provide novel data supporting aerobic preconditioning preceding HIIT as an IL-6 (and possibly hsCRP) lowering intervention in previously sedentary older men. The change in IL-6 and hsCP in SED was largest from A to B (i.e., during preconditioning), but 6weeks of HIIT maintained this reduction despite reduced time commitments. High-intensity aerobic training and high-intensity resistance training have been shown to ameliorate inflammaging; however, the intensity and volume of these exercise modes are rarely met by the general public. Thus, HIIT may provide an alternative option to the current physical activity guidelines to attenuate inflammaging. HIIT has been perceived as more enjoyable than typically time-consuming aerobic conditioning (Thum et al., 2017), and previous investigations from our group have demonstrated HIIT increases perceptions of health-related quality of life, exercise motives, and VO_2peak_ in older men (Knowles et al., 2015; Herbert et al., 2021). Moreover, using the training intervention within this investigation, we have previously reported a shift in the hormonal milieu toward a more “youthful” profile (Hayes et al., 2017, 2020; Herbert et al., 2017a,b). However, 6weeks aerobic conditioning followed by 6weeks of HIIT cannot be considered to reverse inflammatory changes in sedentary older males since the difference between LEX and SED in IL-6 and hsCRP (Cohen’s *d*≥0.74) was much greater than the change in these biomarkers induced by 12weeks of exercise training (Cohen’s *d*≤0.48). In this context, LEX possess a preferential biochemical profile than SED (Hayes et al., 2015, 2020; Elliott et al., 2017) and this investigation extends these data by reporting lower concentrations of some inflammatory mediators due to LEX habits, which were not reduced further by HIIT.

In conclusion, short-term exercise training can reduce some (IL-6 and possibly hsCRP), but not all (homocysteine) inflammatory mediators in SED men, but not LEX men. Moreover, lifelong exercise appears partly anti-inflammatory in sexagenarian men. Taken together, we propose that exercise habits, rather than age *per se*, is more causative of IL-6 (and possibly hsCRP) concentrations in older men. One area for future research could be to extend these data into the oldest old to determine whether lifelong exercise attenuates inflammaging into the eight decade of life and beyond.

## Data Availability Statement

The raw data supporting the conclusions of this article will be made available by the authors, without undue reservation.

## Ethics Statement

The studies involving human participants were reviewed and approved by University of the West of Scotland. The patients/participants provided their written informed consent to participate in this study.

## Author Contributions

PH, NS, and FG: conceptualization and methodology. LH, PH, NS, and FG: formal analysis, investigation, resources, writing – original draft preparation, writing – review and editing, project administration, and funding acquisition. LH: visualization. NS and FG: supervision. All authors contributed to the article and approved the submitted version.

## Conflict of Interest

The authors declare that the research was conducted in the absence of any commercial or financial relationships that could be construed as a potential conflict of interest.

## Publisher’s Note

All claims expressed in this article are solely those of the authors and do not necessarily represent those of their affiliated organizations, or those of the publisher, the editors and the reviewers. Any product that may be evaluated in this article, or claim that may be made by its manufacturer, is not guaranteed or endorsed by the publisher.
